# Two-Stage Microseismic P-Wave Arrival Picking via STA/LTA-Guided Lightweight U-Net

**DOI:** 10.3390/s26051693

**Published:** 2026-03-07

**Authors:** Jiancheng Jin, Gang Wang, Yuanhang Qiu, Siyuan Gong, Bo Ren

**Affiliations:** 1 Huaneng Qingyang Coal Power Co., Ltd., Qingyang 745000, China; 13830325898@163.com; 2 School of Mines, China University of Mining and Technology, Xuzhou 221116, China; ts23020048a31@cumt.edu.cn; 3 School of Computer Science and Technology/School of Artificial Intelligence, China University of Mining and Technology, Xuzhou 221116, China; 4 State Key Laboratory of Safe Mining of Deep Coal and Environmental Protection, Huainan Mining (Group) Co., Ltd., Huainan 232000, China; renbocumt@163.com

**Keywords:** microseismic monitoring, P-wave arrival picking, STA/LTA, lightweight U-Net, two-stage picking, deep learning, mining-induced seismic events

## Abstract

Accurate picking of microseismic P-wave arrival times is essential for the localization and monitoring of mining-induced seismic events. Conventional Short-Term Average/Long-Term Average (STA/LTA) detectors, while computationally efficient, are highly susceptible to noise interference. Conversely, deep learning approaches exhibit superior noise robustness but often involve substantial computational redundancy and compromised real-time performance. To address these limitations, we propose a novel two-stage picking framework that integrates STA/LTA with a lightweight U-Net, enabling rapid preliminary detection followed by fine-grained refinement. In the first stage, STA/LTA rapidly scans continuous waveforms to identify candidate windows potentially containing P-wave arrivals. In the second stage, a lightweight U-Net performs sample-level regression within each candidate window to refine arrival-time estimates with high precision. This coarse-to-fine paradigm effectively balances computational efficiency and picking accuracy. Experimental validation on 500 Hz microseismic data acquired from a coal mine in Gansu Province demonstrates that the proposed method achieves a hit rate of 63.21% within a tolerance window of ±0.01 s. This represents performance improvements of 25.42% and 40.47% over convolutional neural network (CNN) and STA/LTA methods, respectively, while reducing the mean absolute error to 0.0130 s. Furthermore, the model exhibits consistent performance on independent test sets, confirming its generalization capability and noise robustness. By combining the computational efficiency of STA/LTA with the representational power of deep learning, the proposed approach demonstrates significant potential for real-time industrial deployment.

## 1. Introduction

Microseismic monitoring serves as a fundamental technology for predicting and mitigating dynamic disasters in underground mining operations. Source localization accuracy relies heavily on the precision of P-wave arrival-time picking [1,2,3]. Even minor picking errors can propagate into significant localization deviations, thereby compromising the reliability and response speed of early warning systems. Consequently, achieving high-precision, low-latency automatic P-wave picking under strong noise interference remains a critical challenge in microseismic signal processing [4,5,6,7].

Among conventional methodologies, the Short-Term Average/Long-Term Average (STA/LTA) algorithm has been extensively adopted owing to its straightforward implementation and computational efficiency [8,9,10,11]. Beyond the classical STA/LTA algorithm, researchers have developed various advanced signal processing techniques to improve picking accuracy in noisy environments. Methods utilizing multiscale wavelet analysis have proven effective for precise onset detection on single-component recordings [12], while hybrid frameworks combining wavelet denoising with kurtosis-based pickers have demonstrated enhanced robustness in low signal-to-noise ratio (SNR) environments [13]. A comprehensive appraisal of these mathematical tools, ranging from transform-domain to statistical approaches for downhole microseismic data, is provided by Akram and Eaton [14]. However, the STA/LTA method is inherently constrained by empirically determined thresholds, rendering it susceptible to false triggers and missed detections under low SNR conditions. This susceptibility compromises picking accuracy, limiting the algorithm’s applicability in complex operations requiring high precision [15,16]. With the advent of deep learning, architectures such as convolutional neural networks (CNNs), recurrent neural networks (RNNs), and attention mechanisms have been increasingly applied to seismic signal processing, demonstrating marked superiority over traditional methods in terms of noise robustness and picking accuracy [17]. Nevertheless, prevailing deep learning approaches often employ end-to-end full-waveform prediction, resulting in substantial computational overhead and reduced real-time performance. Furthermore, the lack of effective integration with conventional triggering mechanisms leads to computational redundancy and inefficient resource utilization.

To address these limitations, we propose a two-stage P-wave arrival-time picking framework that couples STA/LTA-based preliminary detection with lightweight U-Net refinement. In the first stage, the STA/LTA algorithm scans continuous waveforms to identify candidate arrival points and extract localized windows, thereby avoiding redundant analysis of entire waveforms. In the second stage, a lightweight U-Net architecture performs fine-grained regression within these extracted windows, achieving an optimal balance between computational efficiency and prediction accuracy. The STA/LTA provides a coarse trigger to reduce the computational burden, while the U-Net ensures high-precision refinement to compensate for the limitations of conventional methods under low-SNR conditions. This coarse-to-fine paradigm synergizes traditional algorithms with deep learning, offering strong engineering practicability and extensibility for real-time monitoring systems. Our contributions are summarized as follows:

- A synergistic two-stage picking methodology is proposed that integrates conventional energy-ratio triggering with deep learning models, effectively balancing real-time performance with picking precision.
- An efficient local window extraction mechanism is designed to reduce computational redundancy while enhancing the model’s capacity to capture fine-grained waveform variations via a focusing strategy.
- Comprehensive experiments on real-world microseismic data and independent test sets demonstrate that the proposed method significantly outperforms conventional algorithms in accuracy and robustness, exhibiting strong generalization capability suitable for industrial deployment.

The remainder of this paper is organized as follows: Section 2 details the proposed two-stage framework. Section 3 describes the experimental setup, including data processing and model configuration. Section 4 evaluates the results regarding sensitivity, generalization, and comparative performance. Finally, Section 5 presents the conclusions and future research directions.

## 2. Methodology

This study proposes a two-stage framework for microseismic P-wave arrival picking that synergistically integrates STA/LTA-based preliminary detection with U-Net-guided refinement. The STA/LTA algorithm performs a rapid scan of continuous waveforms to identify candidate P-wave arrival times. This process facilitates the swift triggering and preliminary identification of potential events, enabling the extraction of fixed-length local waveform windows centered on the triggered timestamps. Crucially, this stage is optimized for high sensitivity to ensure that the vast majority of seismic events are captured and passed to the subsequent stage, thereby minimizing the risk of missed detections due to low SNR. This strategy preserves real-time processing capabilities while significantly reducing data volume by focusing the analysis strictly on critical signal segments.

**Figure 1:**
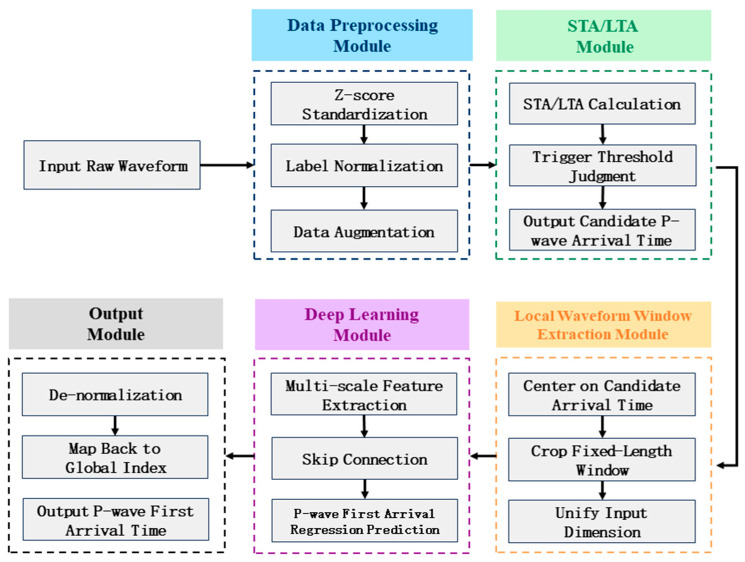
Overall workflow of the proposed methodology.

Subsequently, a lightweight one-dimensional (1D) U-Net is employed to perform fine-grained refinement on the local windows extracted by the STA/LTA. The U-Net architecture utilizes an encoder–decoder structure with skip connections to effectively fuse multi-scale features, thereby enhancing the model’s capacity to capture local waveform characteristics. By combining feature extraction with regression-based prediction, the network achieves precise refinement of P-wave arrival times, significantly improving picking accuracy while maintaining computational efficiency [18,19,20]. The overall workflow of the proposed methodology is illustrated in Figure 1.

### 2.1. STA/LTA Detection and Local Window Extraction

STA/LTA is a widely adopted classical approach for automatic seismic phase picking. Its fundamental principle relies on detecting abrupt amplitude changes within a waveform by computing the ratio of the average signal level in a short-term window to that in a long-term window. The STA captures instantaneous waveform variations, whereas the LTA characterizes the ambient background noise level. A significant increase in the STA/LTA ratio indicates an energy transient, typically corresponding to a P-wave arrival.

In this study, the absolute amplitude of the waveform is employed as the characteristic function for computing STA and LTA values, rather than the conventional squared amplitude [21,22]. The specific calculations are formulated as follows:

(1) $$STA\left(t\right)=\frac{1}{N_{STA}}\sum_{i=0}^{N_{STA}-1}\left|x\left(t-i\right)\right|$$

(2) $$LTA\left(t\right)=\frac{1}{N_{LTA}}\sum_{j=0}^{N_{LTA}-1}\left|x\left(t-j\right)\right|$$

where $N_{STA}$ and $N_{LTA}$ denote the lengths of the short-term and long-term windows, respectively, and $x\left(t\right)$ represents the signal amplitude at time index t. Following the computation of STA and LTA, the ratio R(t)=STA(t)/LTA(t) is calculated to identify potential P-wave arrivals. To ensure numerical stability, the ratio is set to zero whenever the LTA value equals zero.

**Figure 2:**
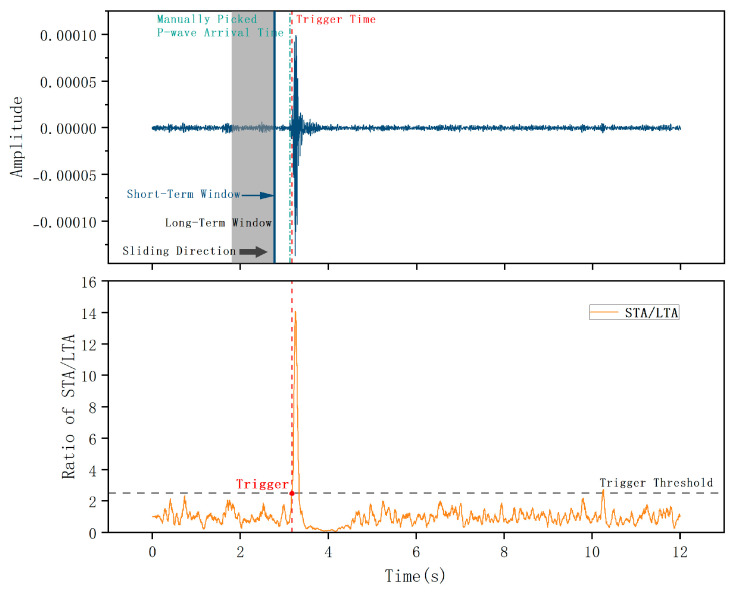
Schematic diagram of the STA/LTA triggering principle.

Compared to conventional energy-squared algorithms, this absolute-value approach effectively captures abrupt waveform transitions while exhibiting lower computational complexity. A P-wave arrival is declared detected when the computed ratio exceeds a predetermined threshold. A schematic illustration of this triggering mechanism is presented in Figure 2.

Since the subsequent U-Net refinement stage relies entirely on the initial windows extracted by the STA/LTA algorithm, minimizing the false negative rate (missed detections) at this stage is critical. A missed detection is defined here as occurring if the STA/LTA algorithm fails to produce any trigger, or a trigger is produced, but the resulting fixed-length window does not contain the true P-wave arrival time.

**Figure 3:**
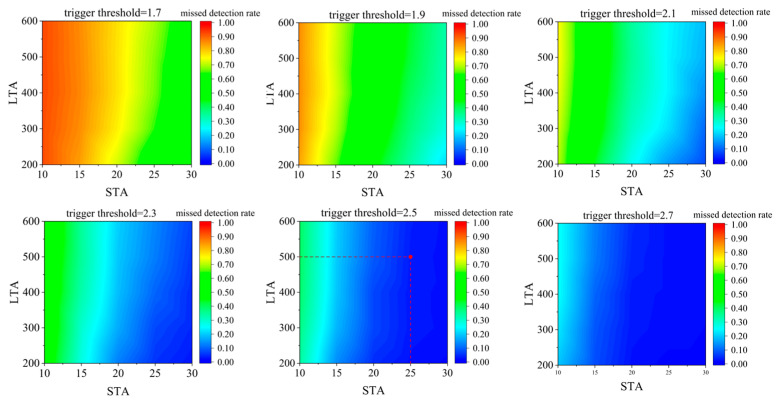
Impact of STA/LTA parameters on detection performance.

To determine the optimal configuration, we conducted a systematic grid search over the parameter space (STA ∈ [10, 30], LTA ∈ [200, 600], trigger threshold ∈ [1.7, 2.7]). The sensitivity analysis results, presented in Figure 3, demonstrate that the missed detection rate stabilizes significantly within specific parameter ranges. Based on these results, we selected a parameter combination of STA = 25, LTA = 500, and Threshold = 2.5. This configuration falls within a high-sensitivity region, yielding a missed detection rate of less than 5% on our validation set, thereby confirming that the vast majority of identifiable events are successfully passed to the U-Net for precise arrival time estimation.

The primary advantages of the STA/LTA algorithm are its high computational efficiency and ease of implementation, making it well-suited for scenarios with stringent real-time requirements. However, the method relies heavily on threshold parameter tuning and is prone to false alarms and missed detections under low signal-to-noise ratio (SNR) conditions, thereby compromising picking accuracy. Consequently, this study employs STA/LTA solely for preliminary detection and local window selection, while a U-Net model is subsequently introduced for fine-grained refinement.

To ensure an effective transition from global scanning to local focusing, we designed an adaptive time-window extraction and standardization strategy based on coarse trigger points. Utilizing the initial arrival time determined by STA/LTA as a reference anchor, fixed-length waveform segments are extracted to construct local time windows focused on the P-wave onset. The design of these windows adheres to two fundamental principles: (1) Information Completeness, which ensures sufficient margins are reserved before and after the trigger point to encompass the true P-wave arrival and contextual features, even given preliminary temporal errors; and (2) Input Standardization, which ensures all time windows are uniformly adjusted to fixed dimensions via zero-padding or truncation to satisfy the deep learning model’s input specifications. This strategy narrows the analysis scope from lengthy continuous waveforms to specific segments containing concentrated feature information, substantially reducing computational overhead. Furthermore, it provides structurally standardized input samples, enabling the network to focus on learning subtle morphological features associated with P-wave onsets, thereby achieving efficient and robust phase picking.

### 2.2. U-Net-Based Fine Regression Refinement

Following preliminary detection and local window extraction, a lightweight 1D U-Net is employed to perform fine-grained regression refinement of the P-wave arrival time. Originally developed for medical image segmentation, the U-Net architecture features a symmetric encoder–decoder structure with skip connections. These mechanisms facilitate the preservation of global contextual information while integrating multi-scale local features, making the architecture particularly well-suited for high-precision signal localization within complex waveforms.

**Figure 4:**
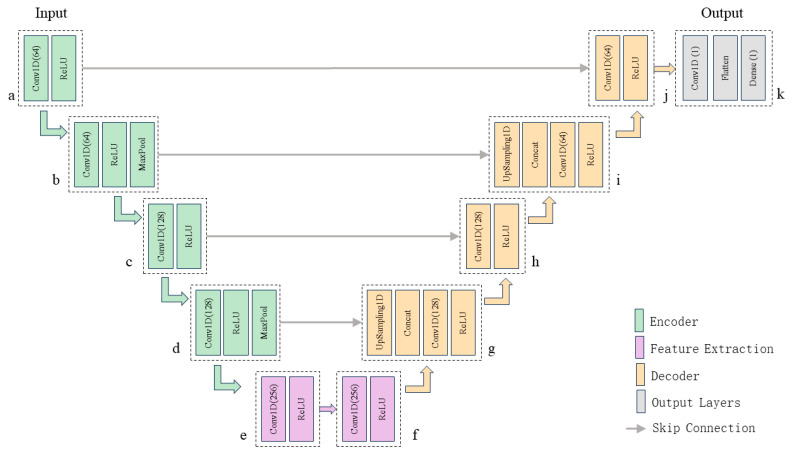
Schematic diagram of the one-dimensional U-Net architecture.

As illustrated in Figure 4, the proposed 1D U-Net model consists of three primary components: an encoder, a bottleneck layer, and a decoder. The input to the network is the standardized waveform sequence extracted via the STA/LTA stage. The encoder performs layer-by-layer convolution and downsampling to extract hierarchical features, progressively reducing temporal resolution while capturing global semantic characteristics. The bottleneck layer consolidates this deep semantic information, and the decoder progressively restores temporal resolution through upsampling. Crucially, skip connections concatenate feature maps from the encoder with corresponding layers in the decoder, achieving multi-scale information fusion and compensating for spatial information lost during downsampling.

The complete network comprises 11 functional modules: modules (a)–(d) constitute the encoder, modules (e)–(f) form the bottleneck layer, modules (g)–(j) comprise the decoder, and module (k) serves as the output layer. The specific operations, parameters, and input–output dimensions for each module are detailed in Table 1.

Through the synergistic design of the U-shaped structure and skip connections, the network simultaneously captures global waveform trends and local morphological details. This dual capability ensures high localization accuracy and robustness, even in noisy environments. Compared with conventional convolutional methods that rely predominantly on local features, the proposed U-Net-based regression model achieves more refined P-wave arrival-time prediction, providing reliable data for subsequent seismic source localization.

## 3. Experimental Setup and Model Training

### 3.1. Experimental Data and Preprocessing

To validate the efficacy of the proposed method, this study utilizes data acquired from an underground microseismic monitoring system deployed in a coal mine in Gansu Province, China. The system employs multi-channel sensors for real-time monitoring at a sampling frequency of 500 Hz. Each waveform recording spans a duration of 12 s, corresponding to 6000 sampling points. To establish a reliable ground truth for model training and evaluation, all data underwent rigorous manual review and precise annotation of P-wave arrival times.

**Figure 5:**
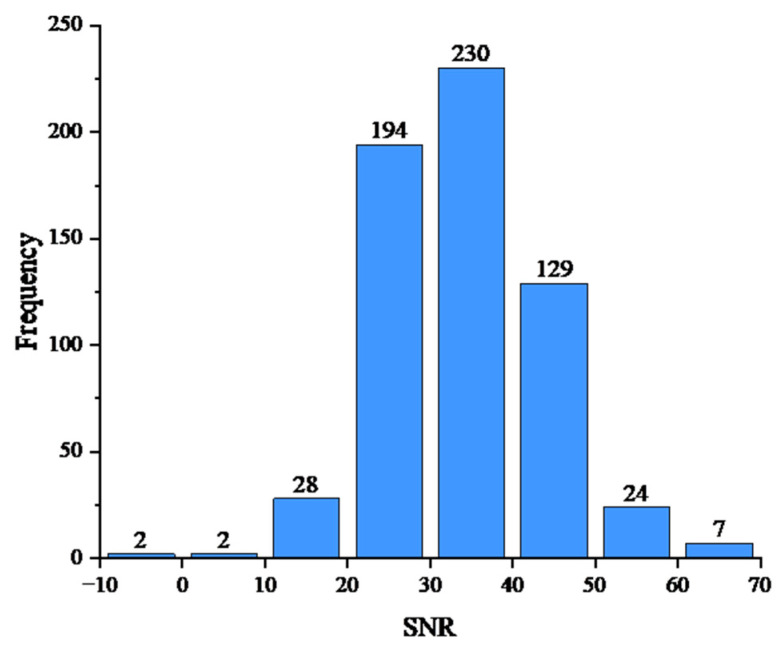
Histogram of waveform SNR distribution.

To assess the data quality and the difficulty of the picking task, we analyzed the SNR distribution of the dataset as shown in Figure 5. The dataset exhibits a mean SNR of 33.99 dB, with values ranging from −9.33 dB to 65.21 dB. As shown in the histogram, the majority of events are concentrated in the 20–50 dB range, indicating a generally high signal quality while still containing low-SNR samples that test the model’s robustness under weak signal conditions.

**Figure 6:**
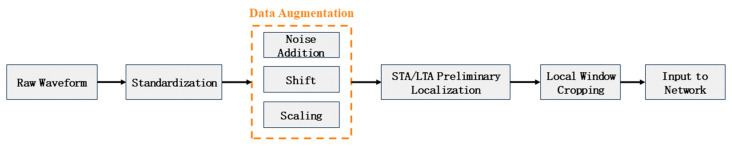
Data processing workflow.

To enhance model stability and generalization capability under complex operational conditions, systematic preprocessing and augmentation techniques were applied to the raw data. The overall workflow, encompassing data standardization, label normalization, and data augmentation, is illustrated in Figure 6.

(1) Data Standardization: Each waveform signal was standardized to zero mean and unit variance (Z-score normalization). This process eliminates the influence of amplitude discrepancies across different sensors and ensures consistent input feature distributions for the neural network.
(2) Label Normalization: The manually annotated P-wave arrival indices were mapped to the normalized interval [0, 1]. This scaling aligns the target values with the output range of the U-Net regression model, facilitating the efficient learning of temporal offset relationships.
(3) Data Augmentation: To improve model robustness against varying noise conditions and enhance adaptability to anomalous samples, multiple data augmentation strategies were implemented to simulate complex field environments. These included:
  - Noise Injection: Gaussian white noise with zero mean and a variance of 0.01 was superimposed on the waveforms to simulate inherent environmental background noise.
  - Temporal Shifting: The entire waveform was randomly shifted within a range of ±10 sampling points to augment the model’s tolerance to minor temporal deviations in signal alignment.
  - Amplitude Scaling: Waveform amplitudes were randomly scaled by factors ranging from 0.9 to 1.1 to accommodate natural amplitude variations observed across different seismic events.

The dataset, comprising both original and augmented samples, was partitioned into training, validation, and test sets at a ratio of 7:1:2. This division ensures diversity and representativeness in the data distribution while effectively mitigating the risk of overfitting. The validation set was employed primarily for hyperparameter tuning and model selection during training to maximize generalization capability.

Consistent with the proposed two-stage framework, the preprocessed data were first subjected to rapid preliminary detection via the STA/LTA algorithm to identify candidate P-wave arrival points. Fixed-length local waveform windows of 200 samples centered on these points were then extracted and fed into the lightweight 1D U-Net for sample-level regression refinement, yielding high-precision P-wave arrival-time predictions.

### 3.2. Baseline Models and Experimental Results

To benchmark the effectiveness of the proposed U-Net model in automatic P-wave identification, this study additionally implemented a classical one-dimensional convolutional neural network (CNN) as a baseline. Unlike the U-Net architecture, the CNN model employs a sequential stacking design comprising multiple convolutional, pooling, and fully connected layers, relying on progressively extracting features through local receptive fields for regression prediction. In these comparative experiments, the STA/LTA algorithm functioned solely as a preliminary detector to generate input windows and was not involved in the subsequent training process. Crucially, all models received identical inputs consisting of local waveform windows generated by STA/LTA, ensuring a fair comparison of feature extraction capabilities.

To guarantee experimental fairness, all models were trained using a consistent set of hyperparameters. The RMSprop optimizer was employed with a learning rate of 0.0001 and a batch size of 32, with training set to a maximum of 200 epochs. Mean Squared Error (MSE) served as the loss function, supplemented by early stopping and adaptive learning rate decay callbacks to prevent overfitting and enhance generalization. All experiments were conducted on an identical hardware platform comprising an Intel Core i5-12600KF processor, an NVIDIA RTX 4060 GPU, and 32 GB of RAM. The software environment consisted of Python 3.9 and TensorFlow 2.11.0.

**Figure 7:**
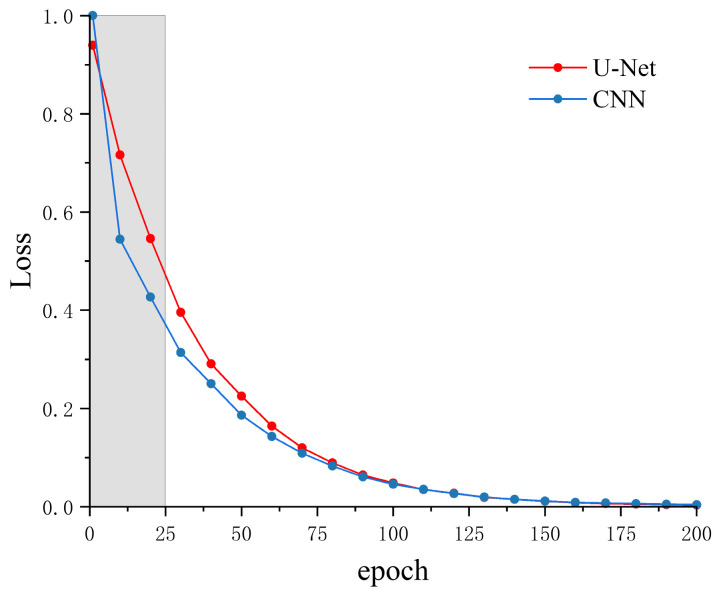
Training loss curves of network models.

**Figure 8:**
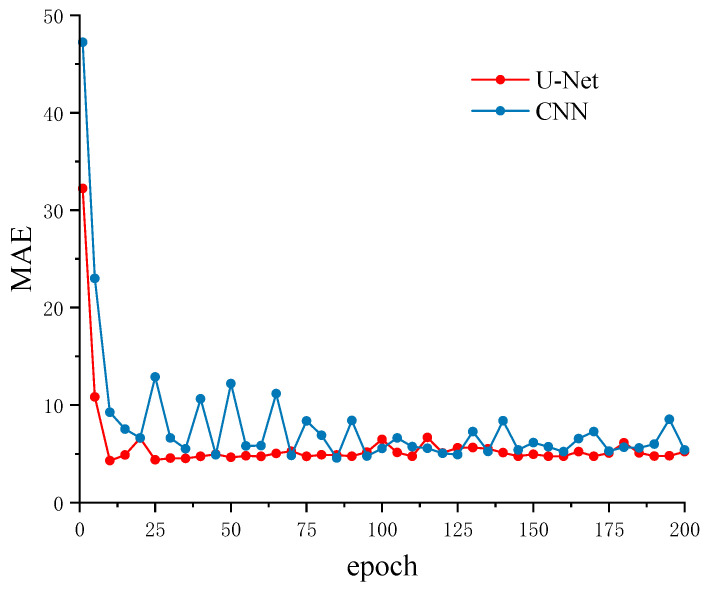
MAE metric evolution curves during training.

Figure 7 and Figure 8 illustrate the loss curves and mean absolute error (MAE) trends, respectively, for the CNN and U-Net models trained on STA/LTA-extracted windows. Regarding overall convergence behavior, the CNN exhibited rapid loss reduction during the initial training phase. However, it subsequently plateaued with notable fluctuations, suggesting a bottleneck in its ability to learn complex local waveform features. In contrast, the U-Net demonstrated more stable convergence throughout the training process, with loss values continuously decreasing and remaining consistently lower than those of the CNN in the mid-to-late stages. Concurrently, the MAE curve for the U-Net exhibited significantly smaller fluctuations, indicating superior learning stability and generalization capability.

It is important to note that since inputs for both models were identical local windows derived from STA/LTA detection, performance differences primarily stem from the models’ inherent feature extraction and representation capabilities rather than data scale or preprocessing. Benefiting from its multi-scale convolution and skip connection mechanisms, the U-Net effectively fuses high-resolution features with deep semantic information during the decoding phase. This architecture strengthens the joint modeling of local transients and global trends, thereby outperforming the sequential CNN architecture in both convergence stability and final accuracy.

In this study, the loss function optimizes the normalized position (range 0–1) of the P-wave arrival within the window. Consequently, the numerical magnitudes are relatively small (approximately 0.003–0.005), making differences between models appear insignificant under this metric. This phenomenon arises from the normalization characteristics of the loss function, which may not fully reflect the actual differences in picking precision. In contrast, MAE, expressed in sampling points, provides a more intuitive and physically meaningful representation of prediction performance.

**Figure 9:**
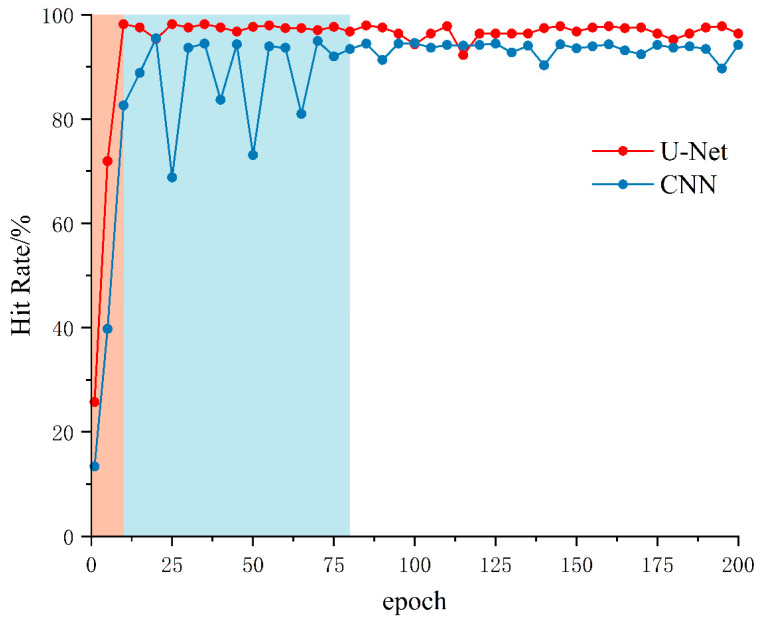
Hit rate curves of two models under a 0.03 s error threshold.

Experimental results indicate that the U-Net achieved a final training loss of 0.0035, lower than the CNN’s 0.0043. Furthermore, the U-Net attained a picking hit rate of 97.8% compared to the CNN’s 94.2%, with MAE values of 5.23 and 5.40 sampling points, respectively. The U-Net also exhibited smaller fluctuation amplitudes and smoother convergence curves. To further evaluate comprehensive model performance, a hit rate metric under a 0.03 s error threshold was introduced to reflect reliability under specific precision requirements. Figure 9 presents the evolution of the hit rate for both models under this threshold across training epochs.

As evident from Figure 9, the U-Net model exhibited rapid hit rate improvement during the early training phase (epochs 0–10), exceeding 95% around epoch 10, and subsequently entering a stable plateau (epochs 10–200) with minimal fluctuations. In contrast, the CNN model displayed pronounced oscillations during the early-to-mid training phases (approximately epochs 10–80), with hit rates fluctuating repeatedly between 70% and 95%. This disparity indicates that the U-Net model possesses distinct advantages in gradient update stability and feature learning robustness.

In summary, the U-Net model outperforms the CNN in terms of convergence speed, stability, and prediction accuracy. These findings validate the effectiveness of the proposed two-stage framework that integrates STA/LTA triggering with U-Net fine regression, demonstrating its favorable engineering applicability and reliability for precise microseismic P-wave arrival picking tasks.

## 4. Model Validation and Result Analysis

To determine the optimal training objective, we briefly investigated the use of MAE, which is often preferred for its robustness to outliers. However, comparative experiments indicated that performance differences between MAE and MSE were marginal. Consequently, MSE was retained for the subsequent experiments in this study due to its favorable convergence stability and its effectiveness in penalizing large error deviations.

### 4.1. Sensitivity Analysis of STA/LTA Trigger Point Offset

In practical underground microseismic monitoring, the STA/LTA algorithm is susceptible to trigger point offsets caused by background noise fluctuations, suboptimal threshold settings, and signal interference. Such offsets can lead to inaccuracies in the positioning of extracted local windows, which may subsequently degrade the precision of P-wave arrival picking in downstream stages [23,24].

This section presents an experimental evaluation using 300 sets of microseismic data. Although these data originate from the same monitoring environment as the training set, they were acquired at independent times and locations to constitute a distinct test set. This setup allows for a rigorous validation of model robustness and generalization capability under varying STA/LTA trigger point offset conditions. All data underwent manual annotation to ensure accurate ground truth for P-wave arrival times.

#### 4.1.1. Experimental Setup

To systematically evaluate the impact of STA/LTA trigger point offsets on picking precision, artificial temporal offsets were introduced to the STA/LTA trigger points of each record. These offsets ranged from −50 ms to +50 ms in increments of 10 ms.

For each offset condition, the trigger point was shifted by the specified magnitude, and a fixed-length window of 200 sampling points was extracted centered on this new position. These windows were then fed into the pre-trained CNN and U-Net models to predict the normalized arrival position. To minimize experimental error, all experiments for each offset magnitude were repeated three times, and the results were averaged. An offset interval of |Δt| ≤ 20 ms was established as the primary region of interest to assess picking stability within permissible temporal deviations.

#### 4.1.2. Experimental Results

Table 2 presents the MAE for both the CNN and U-Net models under different offset conditions. The results indicate that as the offset magnitude increases, the MAE for both models exhibit an upward trend. However, the overall error fluctuations remain limited, suggesting that both models maintain robust performance under most offset conditions.

Specifically, the mean MAE of the CNN model across the entire offset range was 0.03298 s, while that of the U-Net model was 0.03404 s. These values demonstrate no statistically significant difference in precision between the two architectures in this specific test. Within the critical interval of |Δt| ≤ 20 ms, the CNN model achieved an MAE of 0.02095 s and the U-Net model achieved 0.02166 s. Both values are significantly below 0.022 s, indicating that both models are capable of maintaining high picking precision even when the STA/LTA trigger point deviates slightly.

#### 4.1.3. Sensitivity Analysis and Error Comparison

**Figure 10:**
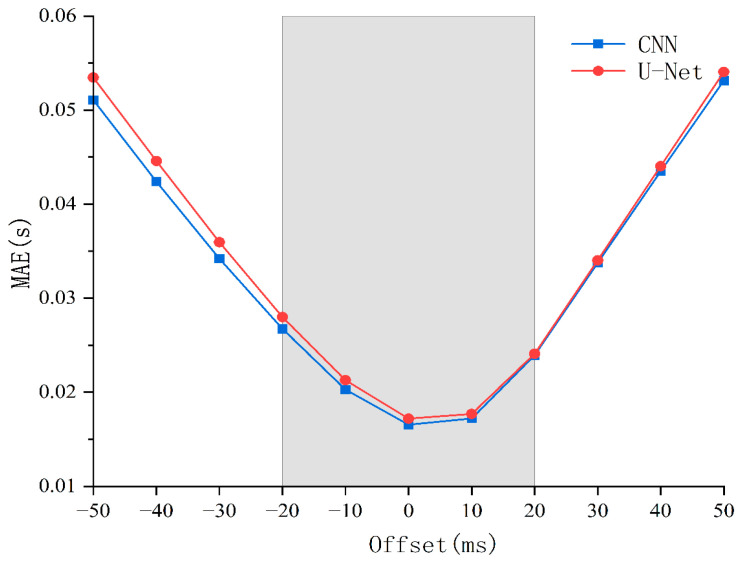
Sensitivity curves of different models to STA/LTA trigger point offset.

Figure 10 illustrates the sensitivity trends of the CNN and U-Net models under different offset magnitudes. The horizontal axis represents the offset magnitude, the vertical axis represents the picking error, and the gray shaded region denotes the |Δt| ≤ 20 ms interval.

The errors for both models increase smoothly as the offset magnitude grows. Within the |Δt| ≤ 20 ms interval, errors remain consistently low (below 0.022 s), indicating that both architectures exhibit strong tolerance to minor offsets.

#### 4.1.4. Error Comparison Within and Beyond ±20 ms Range

To further analyze model error performance under different offset conditions, the offset interval was divided into |Δt| ≤ 20 ms and |Δt| > 20 ms segments, with mean MAE computed separately for each interval.

As shown in Table 3, model errors within the |Δt| ≤ 20 ms interval are significantly lower than those outside the tolerance band. The mean errors for both models within the tolerance band are reduced by over 50% compared to the larger offset range. This indicates that when STA/LTA trigger point errors are controlled within a ±20 ms range, the picking results exhibit notably higher accuracy and consistency.

#### 4.1.5. Analysis and Summary

Based on the comprehensive experimental results, both models demonstrate robust performance under STA/LTA trigger point offset conditions. In the small offset range, the error variations for both CNN and U-Net are relatively gradual, with minimal performance discrepancies. This indicates that both architectures can effectively mitigate the impact of minor trigger point deviations while maintaining high precision and stability.

Across the full offset range, although errors for both models exhibit a positive correlation with offset magnitude, they maintain relatively low error levels within the critical |Δt| ≤ 20 ms interval. These results confirm that both the CNN and U-Net baselines possess favorable precision stability and robustness, validating their suitability for the proposed two-stage framework even in the presence of initial detection errors.

### 4.2. Performance Comparison and Generalization Evaluation

#### 4.2.1. Experimental Objectives and Data Sources

This section evaluates the generalization performance of the proposed method on temporally independent, same-source data and systematically compares the precision of deep learning models against conventional STA/LTA algorithms in P-wave arrival picking tasks. The experiment utilizes the same dataset of 300 underground microseismic records introduced in Section 4.1 as the test set. Crucially, all samples were excluded from the model training and validation phases to ensure the independence and objectivity of the evaluation.

It is important to note that while this section and Section 4.1 utilize identical data, their research objectives differ fundamentally. Section 4.1 focused on model sensitivity to artificial STA/LTA trigger point offsets, testing robustness under simulated conditions. In contrast, this section assesses picking precision and generalization capability under standard operational triggering conditions. Consequently, the findings of these two sections are independent and complementary.

#### 4.2.2. Overall Performance Comparison

Table 4 summarizes the picking results for the three methods across all 300 test samples. The traditional STA/LTA algorithm exhibits inferior overall precision, with an MAE of 0.0545 s and a hit rate below 60% under a 0.02 s error threshold. This performance reflects the inherent limitations of the energy-ratio method when dealing with low SNR or complex waveform morphologies.

In comparison, the deep learning models demonstrate significant improvements in picking precision. The CNN model reduced the mean MAE to 0.0450 s, while the U-Net model achieved 0.0470 s. Both models attained hit rates exceeding 81% within the 0.02 s error tolerance, significantly outperforming the traditional method. This indicates that deep learning architectures possess superior capability in feature extraction and arrival time prediction.

#### 4.2.3. Anomalous Sample Analysis

**Figure 11:**
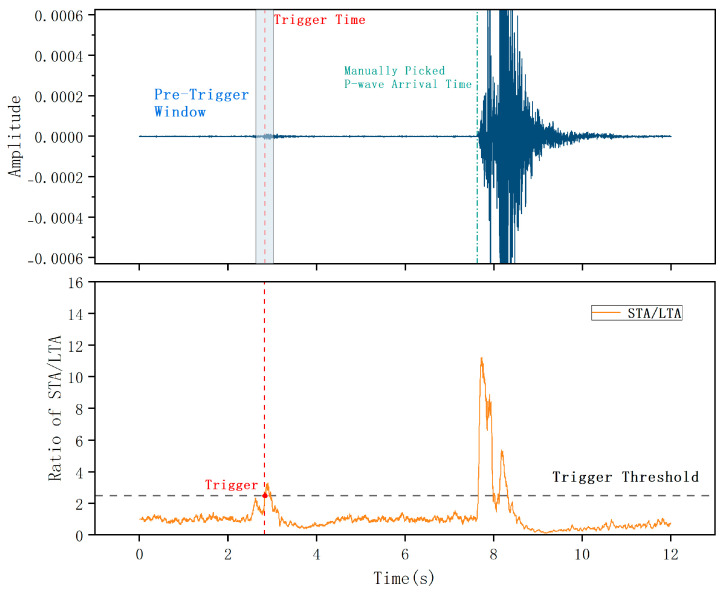
Anomalous sample analysis diagram resulting from premature STA/LTA triggering.

Among the 300 test samples, a single anomalous record exhibiting extreme deviation was identified. In this instance, the STA/LTA trigger time deviated by approximately 4 s from the manually annotated true P-wave arrival. The trigger point was located in the noise interval preceding the main shock energy arrival, clearly outside any reasonable error margin. Figure 11 presents the waveform record and the STA/LTA ratio curve for this sample. The red dashed line indicates the STA/LTA trigger time, the green dashed line represents the ground truth arrival, and the blue shaded region denotes the extracted analysis window.

The anomaly originated from low SNR background conditions, which caused the STA/LTA ratio curve to exhibit a transient spike exceeding the preset threshold, resulting in a premature false trigger. Because the trigger point deviated significantly from the true arrival, the subsequently extracted analysis window fell entirely within the noise segment and contained no valid phase information. Consequently, the deep learning model could not capture meaningful waveform features, leading to a significant prediction error that skewed the overall statistical distribution.

Given that this error stemmed from a false trigger during the STA/LTA preprocessing stage rather than a deficiency in the regression model itself, this sample is treated as an outlier and excluded from the subsequent statistical analysis to ensure the representativeness of the evaluation results.

#### 4.2.4. Performance Evaluation After Exclusion

Following the exclusion of the anomalous sample, the picking precision for all three methods improved significantly. The evaluation results are presented in Table 5. The mean error for the STA/LTA decreased to 0.0226 s, while the CNN and U-Net models achieved mean errors of 0.0149 s and 0.0130 s, respectively. This substantial reduction confirms the distorting effect of the anomalous sample on the initial error statistics.

Regarding hit rate metrics, the deep learning models significantly outperformed the traditional method across multiple error thresholds. Taking the 0.02 s error range as a benchmark, the CNN and U-Net achieved hit rates of 81.61% and 82.61%, respectively, substantially higher than the STA/LTA’s 58.53%. This demonstrates the stronger robustness and consistency of deep learning models under complex operational conditions. Furthermore, the U-Net model achieved a hit rate of 63.21% under the stringent 0.01 s error threshold, superior to the CNN’s 37.79%, highlighting its advantage in high-precision picking tasks.

**Figure 12:**
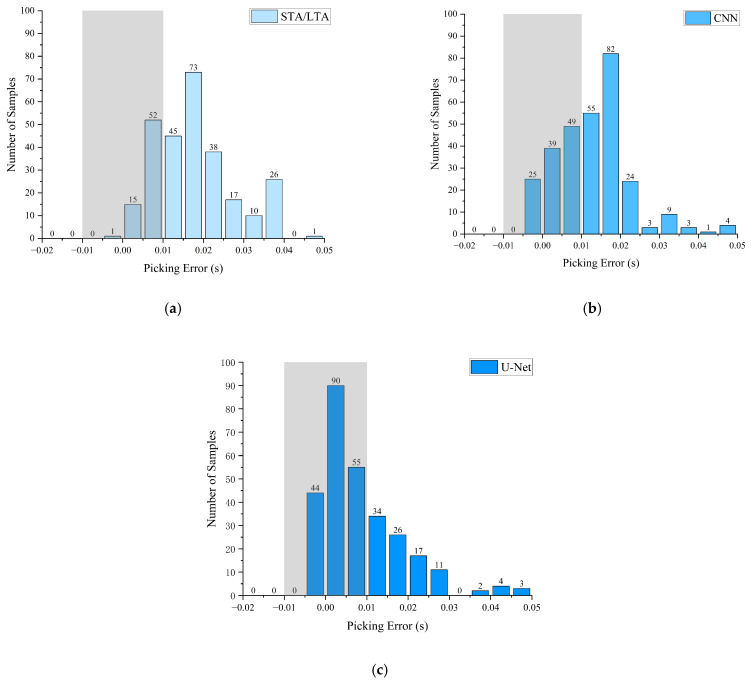
Localization error histograms. (**a**) STA/LTA, (**b**) CNN, (**c**) U-Net.

Figure 12 presents the error distribution histograms for the three methods after excluding the anomalous sample. The STA/LTA method exhibits a dispersed error distribution with pronounced long-tail characteristics, with numerous errors exceeding 0.05 s, indicating substantial fluctuation in precision and insufficient stability.

The CNN model’s errors are predominantly concentrated in the 0.01–0.03 s interval with a relatively symmetric distribution, demonstrating strong prediction stability. However, the distribution still exhibits some tailing, indicating non-negligible deviations in certain samples. In contrast, the U-Net model displays the most concentrated error distribution, featuring a prominent central peak and shorter tails. This indicates higher stability and consistency in capturing P-wave arrival times. The gray rectangular region demarcates the high-precision interval where errors are less than 0.01 s. The U-Net model exhibits the highest sample proportion within this interval, further validating its superiority in high-precision tasks.

In summary, deep learning models, particularly the U-Net, outperform traditional STA/LTA methods in mean error metrics and demonstrate stronger robustness in terms of error distribution concentration and stability.

#### 4.2.5. Comprehensive Analysis

The comparison before and after the exclusion of the anomalous sample highlights the sensitivity of the STA/LTA algorithm to initial trigger accuracy, particularly under low SNR conditions where false triggering amplifies subsequent prediction errors. Although deep learning models possess strong feature extraction capabilities, their performance is contingent upon the input window containing valid phase signals. Therefore, ensuring the accuracy of the initial trigger point remains a critical prerequisite.

Overall, the evaluation results, after excluding the outlier, objectively reflect the true performance of each method. The U-Net model achieves the highest hit rate under the 0.01 s error threshold with the most concentrated error distribution, demonstrating superior generalization capability. These results validate the stability and reliability of the proposed two-stage method on temporally independent data, indicating significant potential for practical deployment.

Furthermore, regarding the adaptability to varying noise structures in practical deployment, the proposed method adopts a transferable strategy. We acknowledge that noise characteristics (e.g., spectral distribution and non-stationarity) can vary significantly across different mining areas due to distinct geological structures and sensor configurations. Therefore, while the processing pipeline (STA/LTA preliminary detection followed by U-Net refinement) remains consistent, the method does not assume invariant noise properties. Instead, it allows for offline fine-tuning or retraining on site-specific datasets when transferred to a new environment with significantly different noise patterns. This approach avoids the computational cost of continuous online retraining while ensuring robustness. Additionally, STA/LTA parameters can be re-optimized for specific site conditions, thereby balancing general applicability with site-specific adaptability.

## 5. Conclusions

This study proposes a novel two-stage framework integrating STA/LTA-based preliminary detection with U-Net refinement to address the challenges in microseismic P-wave arrival picking. The framework utilizes STA/LTA for rapid triggering and local window extraction, drastically reducing data volume, followed by a lightweight U-Net for fine-grained sample-level regression. By synergizing the computational efficiency of traditional algorithms with the robust feature representation of deep learning, the method optimizes both picking speed and precision, particularly under low SNR conditions. Validation results demonstrate that the proposed method significantly surpasses both conventional and deep learning benchmarks in picking accuracy and error minimization. The model exhibits strong generalization on independent datasets and maintains high precision even in the presence of initial trigger offsets, confirming its robustness for practical applications. From an engineering perspective, the proposed approach significantly reduces computational overhead while ensuring high precision, making it highly suitable for deployment in real-time early warning systems for dynamic mining disasters. Future research will focus on integrating attention mechanisms and multi-component signal fusion to enhance feature extraction from complex waveforms. Additionally, we aim to develop adaptive thresholding strategies to optimize STA/LTA triggering and extend the framework to accommodate variable event lengths, thereby broadening its applicability across diverse monitoring systems.

## Figures and Tables

**Table 1:** Parameters and input–output dimensions of network modules.

Module	Operation	Parameters	Input Dimension	Output Dimension
a	Conv1D	filters = 64, k = 3	(200,1)	(200,64)
	ReLU	—	(200,64)	(200,64)
b	Conv1D	filters = 64, k = 3	(200,64)	(200,64)
	ReLU	—	(200,64)	(200,64)
	MaxPool1D	pool = 2	(200,64)	(100,64)
c	Conv1D	filters = 128, k = 3	(100,64)	(100,128)
	ReLU	—	(100,128)	(100,128)
d	Conv1D	filters = 128, k = 3	(100,128)	(100,128)
	ReLU	—	(100,128)	(100,128)
	MaxPool1D	pool = 2	(100,128)	(50,128)
e	Conv1D	filters = 256, k = 3	(50,128)	(50,256)
	ReLU	—	(50,256)	(50,256)
f	Conv1D	filters = 256, k = 3	(50,256)	(50,256)
	ReLU	—	(50,256)	(50,256)
g	UpSampling1D	size = 2	(50,256)	(100,256)
	Concat	—	(100,256) + (100,128)	(100,384)
	Conv1D	filters = 128, k = 3	(100,384)	(100,128)
	ReLU	—	(100,128)	(100,128)
h	Conv1D	filters = 128, k = 3	(100,128)	(100,128)
	ReLU	—	(100,128)	(100,128)
i	UpSampling1D	size = 2	(100,128)	(200,128)
	Concat	—	(200,128) + (200,64)	(200,192)
	Conv1D	filters = 64, k = 3	(200,192)	(200,64)
	ReLU	—	(200,64)	(200,64)
j	Conv1D	filters = 64, k = 3	(200,64)	(200,64)
	ReLU	—	(200,64)	(200,64)
k	Conv1D	filters = 1, k = 1	(200,64)	(200,1)
	Flatten	—	(200,1)	(200,)
	Dense	units = 1	(200,)	(1,)

**Table 2:** Picking error statistics under different offset magnitudes.

Offset (ms)	MAE(s)		Mean Sample Error (pts)		Sample Size
	CNN	U-Net	CNN	U-Net	
−50	0.05313	0.05406	26.56	27.03	300
−40	0.04627	0.04733	23.14	23.67	300
−30	0.03456	0.03529	17.28	17.64	300
−20	0.02312	0.02402	11.56	12.01	300
−10	0.01844	0.01985	9.22	9.93	300
0	0.01655	0.01720	8.27	8.60	300
10	0.01974	0.02012	9.87	10.06	300
20	0.02380	0.02445	11.90	12.23	300
30	0.03302	0.03471	16.51	17.35	300
40	0.04692	0.04780	23.46	23.90	300
50	0.05300	0.05391	26.50	26.96	300

**Table 3:** Comparison of mean MAE within and beyond ±20 ms tolerance band.

Model	|Δt| ≤ 20 ms MAE (s)	|Δt| > 20 ms MAE (s)	Improvement
CNN	0.02095	0.04500	53.4%
U-Net	0.02166	0.04585	52.8%

Note: Improvement = (MAE |Δt| > 20 ms − MAE |Δt| ≤ 20 ms MAE)/MAE |Δt| > 20 ms.

**Table 4:** Test result comparison of different models (including anomalous samples, *N* = 300).

Model	MAE	Hit Rate (%)		
		0.01 s	0.02 s	0.03 s
STA/LTA	0.0545	22.67	58.33	80.33
CNN	0.0450	37.67	81.33	92.33
U-Net	0.0470	63.00	82.33	92.33

**Table 5:** Test result comparison of different models (excluding anomalous samples, *N* = 299).

Model	MAE	Hit Rate (%)		
		0.01 s	0.02 s	0.03 s
STA/LTA	0.0226	22.74	58.53	80.60
CNN	0.0149	37.79	81.61	92.64
U-Net	0.0130	63.21	82.61	92.64

## Data Availability

The datasets presented in this article are not readily available because the data are part of an ongoing study.
